# Ashwagandha (Withania somnifera)-Associated Liver Injury: A Scoping Review of Clinical Characteristics and Safety Considerations

**DOI:** 10.7759/cureus.109764

**Published:** 2026-05-27

**Authors:** Daniel McIntyre, Phong Nguyen, Yujin Kim, Brian Meyer, Michael Salloum

**Affiliations:** 1 Internal Medicine, William Beaumont Army Medical Center, El Paso, USA; 2 Medicine, Texas Tech University Health Sciences Center, El Paso, USA

**Keywords:** ashwagandha, herbal medicine pharmacovigilance, liver injury, plant extracts, scoping review, withania

## Abstract

Background: Ashwagandha (*Withania somnifera*) is a widely used herbal supplement marketed for stress relief and wellness. Although generally perceived as safe, accumulating reports of ashwagandha-associated liver injury have raised concern for hepatotoxic potential.

Materials and methods: We conducted a global scoping review of the peer-reviewed clinical literature to systematically map and synthesize reports of ashwagandha-attributed liver injury. PubMed, Ovid MEDLINE(R) ALL, and MEDLINE Ultimate were searched through December 2025, with supplementary reference list screening. Eligible studies included peer-reviewed case reports and case series describing liver injury associated with single-ingredient ashwagandha products or multi-ingredient formulations containing ashwagandha. Non-peer-reviewed sources and non-English publications were excluded.

Results: Thirteen publications met the inclusion criteria, encompassing 25 patients across multiple global regions. Liver injury typically develops after weeks of use. Cholestatic or mixed biochemical phenotypes predominated, commonly presenting with jaundice and pruritus; purely hepatocellular injury was less frequent. When applied, causality assessments generally classified ashwagandha as a probable or likely cause. Most patients recovered following discontinuation and supportive care, usually over weeks to months. Severe outcomes included one case of acute liver failure requiring transplantation and three deaths among patients with pre-existing cirrhosis who developed decompensation.

Conclusions: The published literature supports the existence of a reproducible clinical syndrome of idiosyncratic, frequently cholestatic, ashwagandha-associated liver injury. Routine assessment of herbal supplement exposure is warranted in unexplained hepatitis or cholestasis, and patients with advanced chronic liver disease should be counseled to avoid ashwagandha. These findings suggest improved supplement traceability and strengthened pharmacovigilance to mitigate herbal-induced liver injury.

## Introduction and background

Introduction

Ashwagandha (*Withania somnifera*), a widely used Ayurvedic botanical, is increasingly consumed worldwide as an over-the-counter dietary supplement for stress, sleep, anxiety, and related wellness goals [1-5]. Although traditionally used for centuries, contemporary commercial preparations are broadly distributed and rapidly expanding in global markets [6-9].

Patients with suspected hepatotoxicity often present through the emergency department (ED) or urgent care with jaundice, pruritus, dark urine, or incidentally detected liver test abnormalities. A national U.S. surveillance analysis estimated that approximately 23,000 ED visits annually were attributed to dietary supplement adverse events [10]. Because drug- and herb-induced liver injury (DILI/HILI) are diagnoses of exclusion, accurate attribution depends on a careful exposure history. However, supplements are frequently omitted unless specifically elicited, increasing the risk of missed etiologies during initial evaluation and counseling. International consensus criteria have standardized clinical-biochemical definitions for DILI, including a phenotype classification based on the relationship between aminotransferase and alkaline phosphatase levels (the R ratio), providing a common framework for case interpretation and reporting [11].

Concerns about the safety of herbal supplements are growing as reports of hepatotoxicity increase, with the first published case linking ashwagandha to liver injury in 2017 [12]. Unlike pharmaceuticals, which undergo rigorous preclinical and clinical evaluation [13], dietary supplements may reach consumers without comparable premarketing safety assessment [14].

Given rising global use and emerging safety signals, there is a need to systematically map the peer-reviewed evidence. This scoping review collates published case reports and case series of ashwagandha-associated liver injury to characterize clinical presentation, diagnostic evaluation, histopathology, causality assessment, and outcomes, and to inform clinicians and regulators regarding potential risk.

Methods

Literature Search Strategy

We searched PubMed, Ovid MEDLINE(R) ALL (1946 to December 12, 2025), and MEDLINE Ultimate through December 2025 for peer-reviewed reports of ashwagandha-associated liver injury, following the Preferred Reporting Items for Systematic Reviews and Meta-Analyses Extension for Scoping Reviews (PRISMA-ScR) framework. The strategy combined Medical Subject Headings (MeSH) and free-text terms for ashwagandha/withania and liver injury/hepatotoxicity (Table 1). A scoping review methodology was selected because the available literature consists predominantly of heterogeneous case reports and case series intended to characterize clinical presentation, assess causality, and report patterns rather than to permit quantitative synthesis or meta-analysis.

**Table 1:** Search query used for specific database

Search no.	Search query
PubMed	
#1	("ashwagandha" OR "withania" OR ("indian" AND "ginseng") OR ("winter" AND "cherry")) AND (("hepatotoxic" OR "hepatotoxicities" OR "hepatotoxicity") OR (("liver" OR "livers") AND ("injury" OR "injured" OR "injuries")) OR (("liver" OR "livers") AND ("wounds" AND "injuries") OR "injurious" OR "injury s" OR "injuryed" OR "injurys" OR "injury")) AND ((humans[Filter]) AND (1940:3000/12/12[pdat]) AND (english[Filter]))
OVID medline	
#2	((ashwagandha or *Withania somnifera* or indian ginseng or winter cherry) and (liver injury or hepatotoxicity or hepatotoxic or hepatocellular injury)).mp. [mp=title, book title, abstract, original title, name of substance word, subject heading word, floating sub-heading word, keyword heading word, organism supplementary concept word, protocol supplementary concept word, rare disease supplementary concept word, unique identifier, synonyms, population supplementary concept word, anatomy supplementary concept word]
#3	Limit 3 to (English language and full text)
MEDLINE ultimate	
#4	(ashwagandha OR *Withania somnifera* OR indian ginseng OR winter cherry) AND (liver injury OR hepatotoxicity OR hepatotoxic OR hepatocellular injury)
#5	Limit 2 to (English language and full text)

Study Selection Process

**Figure 1:**
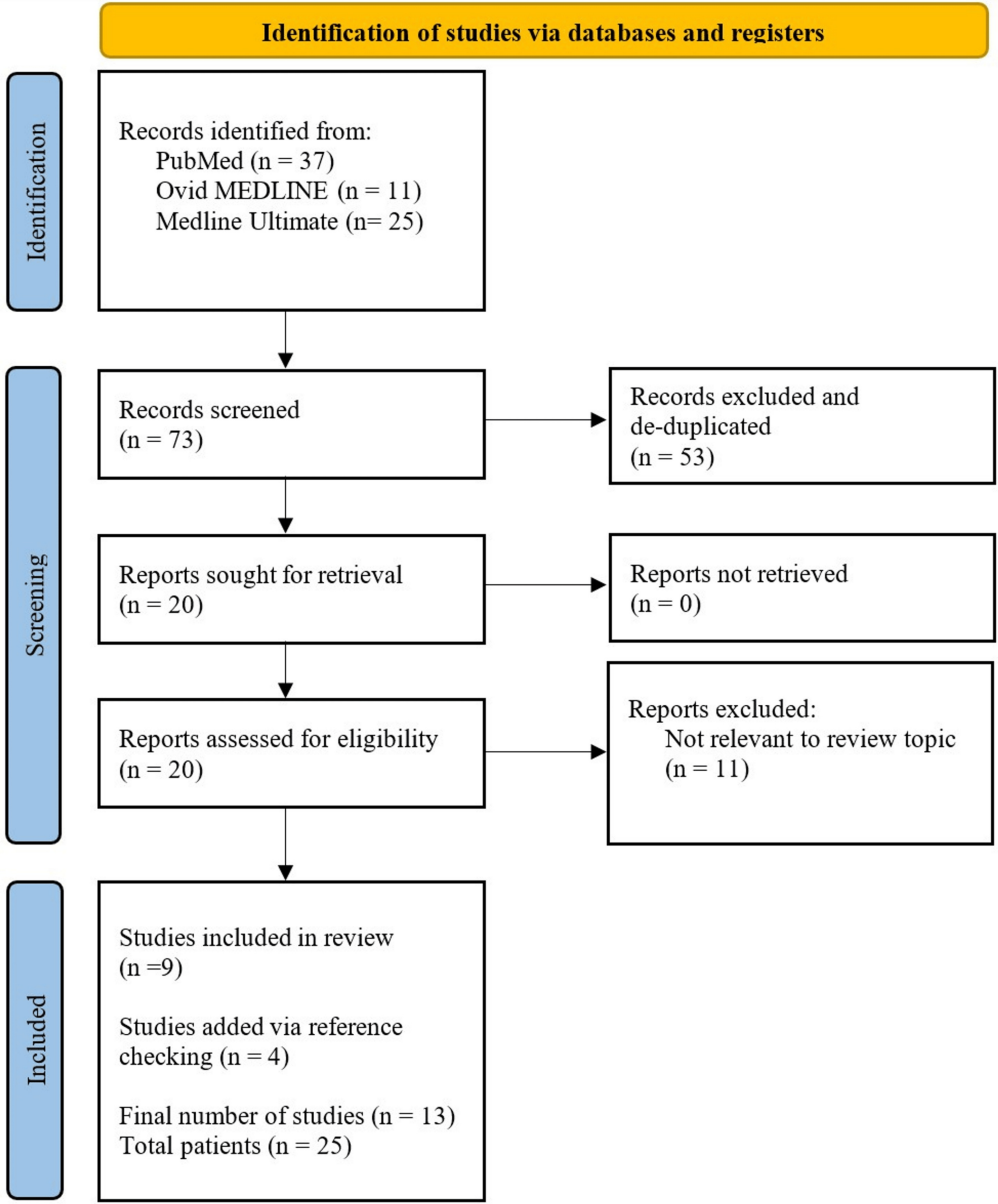
Flow diagram of study selection process

We included peer-reviewed case reports and case series describing liver injury attributed to single-ingredient ashwagandha products or multi-ingredient formulations containing ashwagandha. We excluded non-peer-reviewed sources and non-English publications due to limited methodological vetting, insufficient extractable detail, and translation constraints. Two reviewers independently screened titles/abstracts and subsequently reviewed full texts for eligibility. Discrepancies were resolved by discussion and consensus; when needed, a third reviewer adjudicated disagreements. Formal inter-reviewer agreement statistics (e.g., Cohen’s kappa) were not prospectively calculated. Searches yielded PubMed (n = 37), Ovid MEDLINE (n = 11), and MEDLINE Ultimate (n = 25) records; 73 records were screened, and 20 underwent full-text review. Records underwent citation-manager deduplication followed by manual verification during screening. Although overlap between databases was anticipated, no exact duplicate studies remained after citation normalization and eligibility assessment. Eleven were excluded as irrelevant, leaving nine included studies; four additional studies were identified through reference screening, for a total of 13 studies encompassing 25 patients (Figure 1). We excluded the earliest published study linking ashwagandha to hepatotoxicity because it was published in Japanese and could not be reliably reviewed for inclusion [12].

Data Extraction and Liver Injury-Pattern Classification

For each published study, we abstracted demographics, country, product formulation/dose, duration, and frequency of use, clinical features, laboratory values, diagnostic evaluation, causality assessment, treatment, outcomes, and liver biopsy results when available. Data were charted using a standardized extraction form by two independent reviewers; discrepancies were reconciled by consensus. Liver injury phenotype was recorded as cholestatic, mixed, or hepatocellular as stated by the authors or as “not reported.” Missing data were not imputed and recorded as “not reported.” Cases were not excluded because of missing or "not reported" information. Laboratory values are reported as peak values; when peak values were not explicitly provided, the highest values reported by the authors were used. Findings were synthesized in tabular and narrative form to identify recurrent clinical themes.

## Review

Results

Demographic Characteristics

Published studies encompassed a broad adult age range. The largest proportion occurred in individuals aged 30-39 years (7/25, 28.0%), followed by those aged 40-49 years (6/25, 24.0%). Participants aged 18-29 years and 60-69 years each accounted for 16.0% of cases (4/25 per group). The 50-59 and ≥70-year age strata were least represented (2/25, 8.0% each). With respect to sex, there was a modest male predominance, with males comprising 56.0% of cases (14/25) and females 44.0% (11/25). These statistics are provided in Table 2.

**Table 2:** Demographics, clinical features, outcomes, and publication studies per year of ashwagandha-associated liver injury cases (n = 25)

Category	n	%
Age		
18-29	4	16.0%
30-39	7	28.0%
40-49	6	24.0%
50-59	2	8.0%
60-69	4	16.0%
70+	2	8.0%
Sex		
Male	14	56.0%
Female	11	44.0%
Pattern of liver injury		
Hepatocellular	8	32%
Cholestatic	11	44%
Mixed	5	20%
Not reported	1	4%
Outcome		
Recovered	20	80.0%
Death	3	12.0%
Liver transplant	1	4.0%
Lost to follow-up	1	4.0%
Publication studies per year		
2020	1	7.7%
2021	2	15.4%
2022	1	7.7%
2023	6	46.2%
2024	1	7.7%
2025	2	15.4%

Ashwagandha Formulation and Duration of Use

Across published cases, ashwagandha exposures involved heterogeneous commercial formulations with dosing reported inconsistently. Documented products included branded preparations (NOW Ashwagandha; Nature’s Way), generic single-ingredient capsules (often 450 mg), a root-extract capsule (154 mg), powder/liquid preparations (10-15 g/day, 20 g/day, or 100 mL/day), a regimen switching from a 500-mg extract to 450-mg capsules, and a pastille formulation; however, formulation details were not reported for multiple cases, including within case series.

Exposure duration ranged from very short courses (two days) to several weeks (three to six weeks), months (two to six months), and prolonged use (>1 year), with one report describing extended intake over 540 days. When frequency was specified, use was typically daily (sometimes multiple times per day), but many reports did not provide complete frequency data.

Multi-ingredient exposure was also reported. At least one case involved a “testosterone booster” product containing ashwagandha, and several patients reported concomitant use of other supplements, including Super Male Vitality, spirulina, chlorella, rhodiola, and cerenity [15]. One patient also reported long-term use of Rauwolfia serpentina, a phytotherapeutic product [16].

Patterns in Presentation and Pathology

Patients commonly presented with jaundice-associated symptoms, particularly pruritus and dark urine, often with fatigue, nausea, or malaise. Hyperbilirubinemia ranged from moderate to severe (approximately 5-6 mg/dL to >20 mg/dL), typically accompanied by aminotransferase elevations. Fever, rash, and systemic hypersensitivity features were generally absent.

Across 25 patients, cholestatic injury predominated (11/25, 44%), followed by hepatocellular (8/25, 32%) and mixed (5/25, 20%) patterns; one case was not reported (Table 2). Reports of vanishing bile duct syndrome or irreversible long-term cholestasis have not been published to date.

Diagnostic evaluations generally excluded alternative etiologies: viral hepatitis testing (including EBV/CMV when performed), autoimmune markers, and biliary imaging (ultrasound or MRI/MRCP) were negative or non-diagnostic. Co-exposures were variably reported, with rhodiola considered a possible contributor in one case [15]. In one case series, product analysis did not identify adulterants beyond ashwagandha constituents [15].

Treatments and Outcomes

Outcomes are summarized in Table 2. Encouragingly, most patients experienced complete recovery with supportive management alone. Discontinuation of ashwagandha was the primary intervention; some patients subsequently received adjunctive therapies, including ursodeoxycholic acid, cholestyramine, corticosteroids, N-acetylcysteine (NAC), or plasmapheresis. In nearly all patients, liver tests gradually improved over weeks to months after cessation of ashwagandha. The typical time to full recovery ranged from about one to five months. Only one patient was reported to develop chronic injury: an Indian patient experienced a relapse of hepatitis after initial recovery, consistent with chronic HILI with mildly positive autoimmune markers, but not diagnostic of autoimmune hepatitis [17]. This patient ultimately required a course of corticosteroids, after which his liver tests normalized.

Not all outcomes of ashwagandha-associated liver injury were benign; 16% of published cases resulted in death or required liver transplantation (Table 2). One case of acute liver failure (ALF) attributed to ashwagandha has been reported in the literature [18]. This was a 41-year-old woman in the U.S. who took ashwagandha, along with a progesterone supplement, for two months and developed a fulminant hepatocellular injury leading to ALF. Despite maximal care, she required an urgent liver transplant. Another notable finding in a case series was that, among eight Indian patients with ashwagandha-associated liver injury, three individuals with pre-existing cirrhosis experienced decompensation into acute-on-chronic liver failure and subsequently died despite discontinuation of ashwagandha [17]. All three patients had chronic liver disease (secondary to non-alcoholic fatty liver disease, alcohol, and cryptogenic) and took ashwagandha, hoping to improve their health. The remaining patients in that case series, who had no pre-existing liver disease, recovered with supportive care. Across all published reports to date, no deaths from acute liver injury due solely to ashwagandha have been documented. When fatal outcomes occur, they involve either acute liver transplantation or occur in patients with advanced chronic liver disease.

Liver Biopsy

Liver biopsy was performed in a subset of cases and generally supported a predominantly cholestatic pattern of injury with variable accompanying hepatitis. In Björnsson et al.’s case series, a biopsy obtained 36 days after symptom onset demonstrated acute cholestatic hepatitis with mild-to-moderate inflammation and canalicular cholestasis [15]. In the largest Indian case series, biopsies commonly showed moderate-to-severe cellular and canalicular cholestasis with lymphocytic infiltrates and eosinophils in several patients [17]. Individual reports further illustrate the histologic spectrum, including acute cholestatic hepatitis with confluent necrosis in a patient from the United Kingdom [19], spotty centrilobular necrosis with ceroid-laden macrophages and cholestasis in Germany [16], and a distinct “bland cholestasis” pattern with minimal inflammation in Australia [20]. A potentially confounded U.S. case reported steatohepatitis with stage 2 fibrosis and marked steatosis [21]. In contrast, the single case requiring transplantation demonstrated submassive, pan-zonal (predominantly zone 3) necrosis on explant pathology without fibrosis [18].

Causality Evaluation

Several reports applied formal causality assessment tools. In Björnsson et al.’s case series, the authors reported that the drug-induced liver injury network (DILIN) causality assessment tool was used, which rated ashwagandha as the likely cause in all five cases [15]. Other case reports incorporated the Roussel Uclaf Causality Assessment Method (RUCAM), with most cases falling within the “probable” category. For example, a Libyan patient had a RUCAM score of 7 (probable) [22], and the Polish case [23], U.S. cases [24,25], and the Spanish case [26] were similarly rated “probable” based on RUCAM assessment. Similarly, in Philip and colleagues’ case series, six of eight patients were rated probable and two possible by RUCAM [17]. Other authors relied on rigorous exclusion criteria without a formal score [20,27]. No report described an intentional rechallenge with ashwagandha. A summary of these findings is provided in Table 3.

**Table 3:** Characteristics and key findings of included studies of ashwagandha-associated liver injury ALT: alanine aminotransferase, AST: aspartate aminotransferase, ALP: alkaline phosphatase, RUCAM: Roussel Uclaf Causality Assessment Method, DILIN: drug-induced liver injury network, UDCA: ursodeoxycholic acid, CS: corticosteroids, NR: not reported, * peak values not reported, ^ unit converted from μmol/L to mg/dL

Citation	Age/sex	Formulation	Duration and frequency of use	ALT	AST	ALP	Bilirubin	R-factor	Liver injury pattern	Causality (RUCAM, DILIN)	Treatment	Outcome
Almuzghi et al. (2024) [22]	22 F	Ashwagandha capsule 450 mg	2 days, three tablets a day	315 IU/L	104 IU/L*	150 IU/L	12.85 mg/dL	8.6	Hepatocellular	RUCAM 7 (probable)	UDCA	Recovery in 2 months
Björnsson et al. (2020) [15]	45 M	NOW ashwagandha	NR	345 U/L	226 U/L	279 U/L	14.4 mg/dL	3.3	Mixed	DILIN (definite)	NR	Recovery in 5 months
	24 M	NOW ashwagandha	NR	300 U/L	177 U/L	159 U/L	13.9 mg/dL	3.1	Mixed	DILIN (highly likely)	NR	Lost to follow up
	62 F	NOW ashwagandha	NR	261 U/L	226 U/L	241 U/L	7.5 mg/dL	1.4	Cholestatic	DILIN (probable)	NR	Recovery in 3 months
	61 F	Nature’s Way ashwagandha	NR	580 U/L	334 U/L	187 U/L	5.9 mg/dL	2	Cholestatic	DILIN (possible)	NR	Recovery in 2 months
	21 M	Ashwagandha	NR	317 U/L	235 U/L	274 U/L	8 mg/dL	2.7	Mixed	DILIN (highly likely)	NR	Recovery in 3 months
Bokan et al. (2023) [27]	36 M	Ashwagandha capsule 450 mg	6 months, three tablets a day	1396 IU/L*	439 IU/L*	432 IU/L*	2.63 mg/dL*^	11.1	Hepatocellular	RUCAM 7 (probable)	NR	Recovery in 2 months
	30 F	Ashwagandha capsule 450 mg	45 days, 1 tablet a day	111 IU/L*	75 IU/L*	147 IU/L*	12.57 mg/dL*^	2.6	Mixed	RUCAM 7 (probable)	NR	Recovery in 2 months
Casiano-Manzano et al. (2025) [26]	33 M	Ashwagandha	NR	NR	NR	NR	21.1 mg/dL	NR	Cholestatic	RUCAM 8 (probable)	CS	Recovery (unknown duration)
Ireland et al. (2021) [19]	39 F	Herbal capsule containing ashwagandha root extract 154 mg	6 weeks, 1 tablet a day	1514 IU/L*	NR	184 IU/L	19.12 mg/dL^	26.7	Hepatocellular	RUCAM 8 (probable)	UDCA	Recovery in 14 days
Lubarska et al. (2023) [23]	23 M	NR	NR	490 U/L*	234 U/L*	227 U/L*	28.13 mg/dL	7.98	Hepatocellular	RUCAM 6 (probable)	CS and UDCA plasma exchange	Recovery in 3.5 months
Philips et al. (2023) [17]	75 M	NR	30 days, 20 grams/day	66 IU/L*	107 IU/L*	105 IU/L*	10.4 mg/dL*	1.3	Cholestatic	RUCAM 8 (probable)	NR	Death
	31 F	NR	14 days, 10-15 grams/day	736 IU/L*	598 IU/L*	241 IU/L*	2 mg/dL*	5.6	Hepatocellular	RUCAM 6 (probable)	NR	Recovery in 45 days
	34 M	NR	60 days, 10-15 grams/day	409 IU/L*	647 IU/L*	199 IU/L*	3.6 mg/dL*	5.3	Hepatocellular	RUCAM 8 (probable)	CS	Recovery in 30 days
	58 M	NR	94 days, 500mg daily	159 IU/L*	64 IU/L*	354 IU/L*	21.3 mg/dL*	1.3	Cholestatic	RUCAM 7 (probable)	CS	Recovery in 60 days
	50 F	NR	540 days, 100ml/day	188 IU/L*	235 IU/L*	893 IU/L*	16.1 mg/dL*	0.6	Cholestatic	RUCAM 5 (possible)	NR	Recovery in 95 days
	39 M	NR	30 days, NR	97 IU/L*	47 IU/L*	59 IU/L	11.4 mg/dL*	4.9	Mixed	RUCAM 5 (possible)	NR	Death
	68 M	NR	60 days, NR	63 IU/L*	100 IU/L*	128 IU/L*	24.3 mg/dL*	1.5	Cholestatic	RUCAM 8 (probable)	CS	Recovery in 56 days
	47 M	NR	17 days, NR	354 IU/L*	270 IU/L*	209 IU/L*	31.5 mg/dL*	5.1	Hepatocellular	RUCAM 7 (probable)	NR	Death
Pusec et al. (2022) [21]	43 F	NR	1 year, daily	70 IU/L	195 IU/L	422 IU/L	14.3 mg/dL	NR	Cholestatic	NR	NR	Recovery (unknown duration)
Tóth et al. (2023) [16]	65 F	NR	4 weeks, NR	82 U/L	51 U/L	299 U/L	19.15 mg/dL	NR	Cholestatic	NR	Cholestyramine	Recovered in 3 months
Vazirani et al. (2023) [24]	48 M	TestBoost	2 days, NR	152 IU/L	127 IU/L	140 IU/L	13.6 mg/dL	NR	Cholestatic	RUCAM 6 (probable)	NR	Confounded by alcohol use disorder
Zhuang et al. (2025) [20]	77 F	Ashwagandha pastille form	3 weeks, NR	145 IU/L*	67 IU/L*	270 IU/L*	7.72 mg/dL*^	NR	Cholestatic	NR	NR	Recovery in 4 months
Suryawanshi et al. (2023) [18]	41 F	NR	2 months, NR	3400 U/L	2500 U/L	102 U/L	24.6 mg/dL	> 5	Hepatocellular	RUCAM (score NR, possible)	N-Acetylceysteine and liver transplant	Require liver transplant
Weber et al. (2021) [25]	40 M	Ashwagandha extract 500 mg, then NOW ashwagandha 450 mg	>1 year, NR	NR	NR	NR	25.4 mg/dL	NR	NR	RUCAM 6 (probable)	NR	Recovered (unknown duration)

Discussion

This global scoping review synthesizes the peer-reviewed clinical literature on ashwagandha-associated liver injury. It identifies a recurring clinical pattern that may aid in recognizing possible HILI. Across 13 publications encompassing 25 patients, most cases presented after a latency of weeks with a predominantly cholestatic or mixed biochemical phenotype and prominent jaundice and pruritus, followed by a prolonged recovery trajectory after cessation. Although most patients recovered with supportive care, severe outcomes, including ALF requiring transplantation and deaths among patients with pre-existing cirrhosis, demonstrate that ashwagandha-associated hepatotoxicity, while uncommon in the published record, can be clinically consequential and should be incorporated into routine diagnostic reasoning for unexplained liver injury.

For general internal medicine clinicians, the principal implication is practical: Ashwagandha and other dietary supplements should be treated as bona fide exposures in medication reconciliation and in the evaluation of liver injury. The cases summarized here repeatedly illustrate that diagnosis hinges on deliberate history taking (including product formulation, timing, and co-exposures), exclusion of competing etiologies, and prompt discontinuation, with early triage for severity. The consistency of clinical phenotypes across geographically distinct reports and the frequent “probable/likely” causality assessments further support that this is not an isolated phenomenon but a reproducible pattern of DILI.

Pharmacovigilance Signals Support Without Quantifying Population Risk

We queried the Food and Drug Administration Adverse Event Reporting System (FAERS) Public Dashboard [28] on 8 December 2025 using product/ingredient terms “ashwagandha” and “*Withania somnifera*.” Reports were summarized at the case level and tabulated by year. Similarly, we queried World Health Organization VigiAccess [29] on 8 December 2025 using the ingredient-based entry for *Withania somnifera* (with “ashwagandha” as a cross-check) and extracted aggregated counts by year.

**Figure 2:**
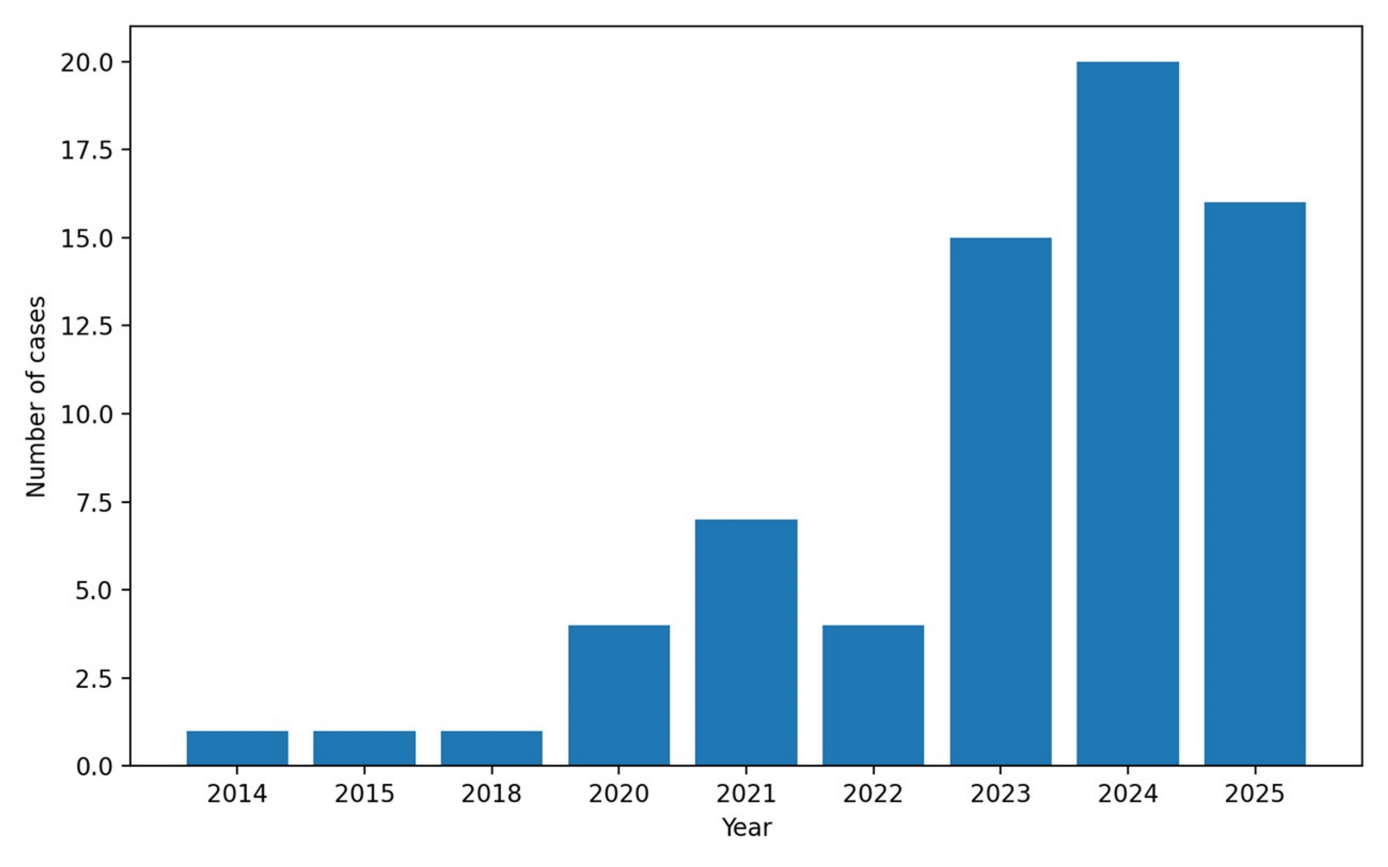
Annual FDA FAERS reports of ashwagandha-associated liver injury FDA FAERS: Food and Drug Administration Adverse Event Reporting System

**Figure 3:**
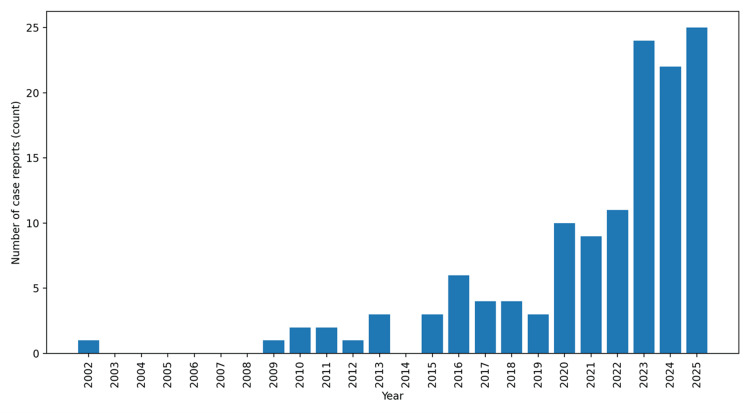
Annual WHO VigiAccess reports of ashwagandha-associated hepatic adverse events WHO: World Health Organization

In the FAERS Public Dashboard, multiple reports of hepatic adverse events list ashwagandha-containing products as primary or secondary suspect agents. Reports have increased in recent years, paralleling the expansion of the U.S. dietary supplement market (Figure 2). However, spontaneous reporting systems are subject to important limitations, including selective and stimulated reporting, possible duplicate or follow-up submissions, and the absence of reliable exposure denominator data, which precludes estimation of true incidence or comparative risk. WHO VigiAccess similarly documents hepatic reports attributed to *Withania somnifera* across multiple regions and adult age groups (Figure 3). These pharmacovigilance data were interpreted as supportive safety signals rather than confirmed causality assessments. Publicly available reports were reviewed at the case level when feasible to minimize obvious duplicate or follow-up entries, although complete deduplication cannot be guaranteed. Reports involving multi-ingredient formulations were retained because commercial ashwagandha products are frequently combined with other supplements; however, such co-exposures limit definitive attribution of causality to ashwagandha alone. It should be noted, however, that spontaneous reporting systems such as FAERS and VigiAccess are subject to substantial underreporting, selective reporting, and stimulated reporting following media coverage or regulatory attention; they also lack a true exposure denominator, precluding estimation of incidence or quantification of comparative risk. Reports may be further confounded by multi-ingredient supplement formulations, co-exposures, and variable product quality, and duplicate or follow-up submissions can inflate counts despite case-level handling. Nevertheless, the convergence of consistent clinical phenotypes in peer-reviewed case reports with parallel national and international pharmacovigilance signals provides convergent validity supporting a reproducible safety concern.

The global distribution of published cases suggests that risk is not confined to a single population or manufacturer. The lack of peer-reviewed hepatotoxicity reports before 2017, despite long-standing traditional use in India and earlier safety signals in spontaneous-reporting pharmacovigilance databases, may reflect differences in preparation (traditional lower-dose root preparations vs. modern concentrated extracts), expanding global exposure, and historical under-recognition and under-reporting of HILI. As clinical awareness has increased, case detection has risen, as evidenced by recent series from India.

Biological Plausibility and Product Heterogeneity Are Credible but Mechanisms Remain Unproven

The mechanistic basis of ashwagandha-associated liver injury remains incompletely defined; however, available experimental evidence supports biological plausibility and highlights the likely importance of product heterogeneity. In vitro studies indicate that select withanolides, including withanone, can induce cellular stress and DNA damage, particularly under conditions of reduced antioxidant capacity, consistent with a reactive metabolite-mediated susceptibility model [30]. Complementary work demonstrates that ashwagandha extracts can modulate cytochrome P450 activity and produce dose-dependent cytotoxicity in primary human hepatocytes, supporting the plausibility of herb-drug interactions and differential toxicity across preparations [31].

Clinically, the predominance of cholestatic or mixed phenotypes suggests downstream perturbations in bile formation or transport, but existing data do not establish a single culpable constituent or a definitive transporter-mediated mechanism. One plausible explanation for the predominance of cholestatic presentations is that withanolide-containing extracts may interfere with hepatobiliary transporter function or bile acid homeostasis, thereby impairing bile flow rather than causing isolated hepatocellular necrosis. Modern commercial products often contain concentrated extracts with variable composition (plant part, extraction solvent/method, and withanolide content), which may diverge from historical preparations and plausibly contribute to variable exposure and risk. Mechanistic attribution will therefore require linking chemically characterized commercial products to reproducible hepatotoxic phenotypes in experimental systems, rather than extrapolating from non-standardized extracts.

Idiosyncratic Susceptibility With Disproportionate Harm in Advanced Liver Diseases

Across published case studies and case series, no consistent demographic risk factor (age or sex) or clear dose-response relationship is apparent, supporting an idiosyncratic susceptibility paradigm rather than predictable intrinsic hepatotoxicity. Most patients reported using typical commercial doses and developed injury after a latency of weeks to months, and the recurrent cholestatic or mixed phenotype across unrelated patients in multiple regions is consistent with an idiosyncratic pattern of HILI.

At the same time, outcomes suggest a clinically meaningful risk gradient. Severe outcomes appeared concentrated among patients with limited hepatic reserve; in the largest series, the most serious cases occurred in individuals with pre-existing cirrhosis who developed acute-on-chronic liver failure and subsequently died [17]. Although case reports cannot quantify attributable risk, these patterns justify conservative counseling for patients with cirrhosis or advanced chronic liver disease. Avoidance of ashwagandha is prudent given the potential for decompensation and the absence of premarketing safety assurance for concentrated, heterogeneous commercial preparations.

Co-exposures represent an important and incompletely characterized modifier of risk. Multi-ingredient formulations, concomitant prescription medications (including hormonal agents), and alcohol exposure may contribute via additive hepatotoxicity or metabolic interactions, yet current reporting is insufficient to define specific interaction patterns. To strengthen causal inference and enable stratified risk assessment, future reports should systematically document all co-exposures and provide complete product details, including ingredient lists, brand and batch/lot identifiers, and, when feasible, independent chemical characterization.

Clinical Recognition and Management Should Prioritize Exposure History, Severity Triage, and Avoidance of Rechallenge

Although ashwagandha is widely marketed as an over-the-counter supplement for stress relief and other wellness benefits, the cases summarized here reinforce that clinicians should treat ashwagandha and other herbal/dietary supplements as bona fide medication exposures during medication reconciliation and in the diagnostic work-up of acute hepatitis, mixed-pattern injury, or cholestatic hepatitis with jaundice and pruritus. Standard DILI and herbal/dietary supplement guidance emphasizes that diagnosis hinges on careful chronology, exclusion of competing etiologies, and documentation of the rechallenge response after immediate discontinuation of the suspected agent(s) [32,33]. Accordingly, our proposed approach prioritizes (1) detailed exposure ascertainment (brand/manufacturer, formulation, dose, start/stop dates, co-ingestants, and lot/batch identifiers), (2) prompt cessation of ashwagandha and other nonessential supplements, and (3) early biochemical pattern classification and targeted evaluation (viral hepatitis, autoimmune markers when appropriate, and biliary imaging for cholestatic presentations). Patients should be explicitly counseled to avoid rechallenge and to avoid switching to alternative brands, given formulation heterogeneity and uncertain recurrence risk [32,33].

Management is predominantly supportive and phenotype-directed. For prolonged cholestasis with pruritus, a stepwise antipruritic strategy (typically starting with bile acid sequestrants, followed by second-line agents such as rifampin, opioid antagonists, or sertraline when appropriate) may be considered, balancing efficacy against drug interactions and hepatotoxic potential in patients with active liver injury [34]. Corticosteroids are not routinely indicated in idiosyncratic DILI/HILI. They should be reserved for situations with strong concern for autoimmune hepatitis or autoimmune-like injury, based on the totality of serologies, IgG, and/or histology, or for persistent inflammatory injury despite withdrawal and specialist evaluation [32,33]. In severe or refractory cases, particularly when ALF physiology is developing, extracorporeal support, such as high-volume plasma exchange, has been shown to improve transplant-free survival in ALF populations and may serve as a bridge for selected patients managed in specialized centers [35].

A central practical priority for internal medicine clinicians is early identification of severe hepatocellular injury and suspected ALF, because transfer and transplant evaluation are time sensitive. The American Association for the Study of Liver Diseases guidance recommends that contact with a transplant center and transfer planning be initiated early in the evaluation of patients with ALF [36]. Clinically, this translates to urgent escalation when there is coagulopathy (INR ≥1.5) with any encephalopathy, rapidly worsening synthetic function, hypoglycemia, rising bilirubin with systemic instability, or concern for evolving multiorgan failure [36,37]. In parallel, NAC should be considered early when ALF is suspected, even when acetaminophen exposure is uncertain, because randomized trial evidence demonstrates improved transplant-free survival for patients with early-stage non-acetaminophen ALF treated with intravenous NAC, with an acceptable safety profile [38]. Notably, the single published case of ashwagandha-associated ALF requiring urgent liver transplantation reported NAC administration prior to transplant [18], underscoring that severe outcomes, while uncommon, are plausible and mandate early escalation pathways.

Regulatory Gap and Policy Implications

In the United States, dietary supplements are regulated under a distinct statutory framework from prescription drugs; under the Dietary Supplement Health and Education Act, the FDA generally does not “pre-approve” dietary supplements for safety or effectiveness (or their labeling) before they are marketed, placing primary responsibility on manufacturers to ensure products are not adulterated or misbranded [39]. Although post-market oversight includes mandatory reporting of serious adverse events by the “responsible person” and manufacturing quality requirements under dietary supplement Current Good Manufacturing Practices, these mechanisms do not reliably ensure product comparability, traceability, or early detection of signals of hepatotoxicity [40].

To reduce preventable harm while preserving access, policy levers that are feasible include (1) strengthening adverse event reporting by standardizing required fields with completed product identifiers, batch/lot, plant part, and extraction type and expanding structured reporting beyond “serious” events to improve signal detection; (2) implementing more stringent identity and contaminant testing expectations, including routine third-party verification for botanical identity, purity, and contaminant limits consistent with quality specifications [41]; (3) establishing labeling requirements specific to botanicals that materially affect exposure, such as root vs. leaf, extraction method/solvent, and a quantified withanolide content range, to support traceability and clinically meaningful history taking [42]; and (4) adding targeted warnings for higher-risk groups (patients with cirrhosis or advanced chronic liver disease) and explicit guidance to discontinue use and seek evaluation if jaundice or pruritus develops.

Strengths and Limitations

Strengths of this scoping review include comprehensive database searching, global case capture, and integration of peer-reviewed clinical evidence with a supportive pharmacovigilance context. Limitations reflect the case-based evidence base: variable reporting completeness, heterogeneous formulations, frequent co-exposures, and limited characterization of product chemicals. Publication bias and selective reporting are likely, and exclusion of non-English and non-peer-reviewed reports may have omitted additional cases that could further expound liver injury patterns. Pharmacovigilance databases cannot establish causality or incidence. Despite these limitations, the convergence of consistent clinical phenotypes across geographies and repeated assessments of probable/likely causality support a safety concern relevant to general internal medicine practice.

## Conclusions

Ashwagandha has been associated with acute liver injury worldwide, commonly presenting after weeks of use as a cholestatic or mixed-pattern hepatitis with jaundice, pruritus, and a recovery course that may extend for weeks to months after discontinuation. Clinicians should explicitly assess for ashwagandha and other supplement exposures when evaluating unexplained liver injury, particularly cholestasis, and counsel patients with cirrhosis or advanced chronic liver disease to avoid ashwagandha given the risk of severe decompensation. Further work is needed to clarify incidence, identify host susceptibility factors, and link chemically characterized product constituents to mechanistic pathways. In the interim, the existing case literature provides a safety signal that supports heightened vigilance, improved product traceability, and strengthened regulatory and pharmacovigilance strategies.

## Appendices

**Table 4:** PRISMA-ScR checklist PRISMA-ScR: Preferred Reporting Items for Systematic reviews and Meta-Analyses Extension for Scoping Reviews

Section	Item	PRISMA-ScR checklist item	Reported location
Title			
Title	1	Identify the report as a scoping review.	Title reflects
Abstract			
Structured summary	2	Provide a structured summary that includes (as applicable): background, objectives, eligibility criteria, sources of evidence, charting methods, results, and conclusions that relate to the review questions and objectives.	Abstract section
Introduction			
Rationale	3	Describe the rationale for the review in the context of what is already known. Explain why the review questions/objectives lend themselves to a scoping review approach.	Introduction: ¶ 1-4
Objectives	4	Provide an explicit statement of the questions and objectives being addressed with reference to their key elements (e.g., population or participants, concepts, and context) or other relevant key elements used to conceptualize the review questions and/or objectives.	Introduction: ¶ 2
Methods			
Protocol and registration	5	Indicate whether a review protocol exists; state if and where it can be accessed (e.g., a Web address); and if available, provide registration information, including the registration number.	Methods: ¶ 1
Eligibility criteria	6	Specify characteristics of the sources of evidence used as eligibility criteria (e.g., years considered, language, and publication status), and provide a rationale.	Table 1
Information sources	7	Describe all information sources in the search (e.g., databases with dates of coverage and contact with authors to identify additional sources), as well as the date the most recent search was executed.	Methods: ¶ 1
Search	8	Present the full electronic search strategy for at least 1 database, including any limits used, so it can be repeated.	Table 1
Selection of sources of evidence	9	State the process for selecting sources of evidence (i.e., screening and eligibility) included in the scoping review.	Methods: ¶ 2,3
Data charting process	10	Describe the methods of charting data from the included sources of evidence (e.g., calibrated forms or forms that have been tested by the team before their use, and whether data charting was done independently or in duplicate) and any processes for obtaining and confirming data from investigators.	Methods: ¶ 2,3
Data items	11	List and define all variables for which data were sought and any assumptions and simplifications made.	Methods: ¶ 3
Critical appraisal of individual sources of evidence	12	If done, provide a rationale for conducting a critical appraisal of included sources of evidence; describe the methods used and how this information was used in any data synthesis (if appropriate).	Methods: ¶ 2
Synthesis of results	13	Describe the methods of handling and summarizing the data that were charted.	Methods: ¶ 3
Results			
Selection of sources of evidence	14	Give the number of sources of evidence screened, assessed for eligibility, and included in the review, with reasons for exclusions at each stage, ideally using a flow diagram.	Figure 1 and ¶ 1
Characteristics of sources of evidence	15	For each source of evidence, present characteristics for which data were charted and provide the citations.	Table 3
Critical appraisal of sources of evidence	16	If done, present data from the critical appraisal of the included sources of evidence (see item 12).	Results: ¶ 11
Results of individual sources of evidence	17	For each included source of evidence, present the relevant data that were charted that relate to the review questions and objectives.	Table 3
Synthesis of results	18	Summarize and/or present the charting results as they relate to the review questions and objectives.	Discussion: ¶1-2
Discussion			
Summary of evidence	19	Summarize the main results (including an overview of concepts, themes, and types of evidence available), link to the review questions and objectives, and consider the relevance to key groups.	Discussion: ¶ 1-2
Limitations	20	Discuss the limitations of the scoping review process.	Discussion: ¶ 16
Conclusions	21	Provide a general interpretation of the results with respect to the review questions and objectives, as well as potential implications and/or next steps.	Conclusion: ¶1
Funding			
Funding	22	Describe sources of funding for the included sources of evidence, as well as sources of funding for the scoping review. Describe the role of the funders of the scoping review.	Within the Cureus publication submission
